# Vitexin Mitigates Myocardial Ischemia/Reperfusion Injury in Rats by Regulating Mitochondrial Dysfunction via Epac1-Rap1 Signaling

**DOI:** 10.1155/2021/9921982

**Published:** 2021-06-28

**Authors:** Huanhua Yang, Wei Xue, Caijuan Ding, Cheng Wang, Bohan Xu, Shuo Chen, Binshan Zha, Yuqian Sun, Huaqin Zhu, Junyan Zhang, Liuyi Dong

**Affiliations:** ^1^Department of Pharmacology, Key Laboratory of Chinese Medicine Research and Development of State Administration of Traditional Chinese Medicine, Anhui Medical University, Hefei, Anhui, China; ^2^Department of Medical, Tongling Polytechnic, Tongling, Anhui, China; ^3^Department of Physical Diagnostics, First Clinical Medical College, Anhui Medical University, Hefei, Anhui, China; ^4^Department of Vascular and Thyroid Surgery, Department of General Surgery, The First Affiliated Hospital of Anhui Medical University, Hefei, Anhui, China; ^5^Laboratory of Molecular Biology and Department of Biochemistry, Anhui Medical University, Hefei, Anhui, China

## Abstract

Revascularization is an effective therapy for rescuing myocardial tissue after ischemic events. However, the process of reperfusion can lead to more severe cardiomyocyte damage, called myocardial ischemia-reperfusion (I/R) injury (MIRI). We have previously shown that vitexin (VT) (a flavonoid compound derived from natural products) protects against MIRI; however, the exact mechanisms underpinning this effect require further elucidation. This study is aimed at elucidating the protective mechanism of VT in inhibiting ischemic myocardial mitochondrial dysfunction and reducing cardiomyocyte apoptosis by regulating Epac1-Rap1 signaling. Isolated rat hearts were subjected to MIRI in a Langendorff perfusion system, and H9c2 cells were subjected to hypoxia/reoxygenation (H/R) in vitro. Our analyses show that during I/R, Epac1 expression was upregulated, left ventricular dysfunction deteriorated, mitochondrial dynamics were disrupted, and both myocardial cells and tissues exhibited apoptosis. Furthermore, administration of 8-CPT (an Epac agonist) exacerbated cardiomyocyte injury and mitochondrial dysfunction. Interestingly, suppressing the function of Epac1 through VT or ESI-09 (an Epac inhibitor) treatment during I/R reduced the myocardial infarct size, cardiomyocyte apoptosis, and reactive oxygen species production; alleviated mitochondrial dysfunction by increasing mitochondrial membrane potential; elevated MFN2 expression; and inhibited Drp1 expression. To our knowledge, our results reveal, for the first time, the mechanisms underlying the protective effect of VT in the myocardium of rats with MIRI. Moreover, we provide a new target and theoretical basis for VT in the treatment of ischemic heart disease.

## 1. Introduction

Ischemic heart disease has long been one of the leading causes of death worldwide, with high morbidity and mortality [1]. Revascularization is the most effective therapy for rescuing ischemic cardiomyocytes, and it can significantly reduce complications and mortality. However, coronary artery occlusion and abrupt recovery of blood flow can lead to more severe cardiomyocyte damage, which is called myocardial ischemia-reperfusion (I/R) injury (MIRI) [2]. Cardiac dysfunction caused by MIRI includes several types of fatal reperfusion injury, no-reflow phenomenon, and perfusion arrhythmia [3, 4]. Although the mechanisms underpinning MIRI have not been fully elucidated, several studies have revealed that it involves a decrease in adenosine triphosphate (ATP) production, elevation of reactive oxygen species (ROS) levels, suppression of mitochondrial membrane potential (MMP), and an overload of Ca^2+^ caused by mitochondrial dysfunction [5–7]. At present, researchers have focused on treating I/R injury with drugs or stem cells. In general, drug intervention has been considered relatively beneficial for the treatment of cardiac ischemia [8], and the cardioprotective effects of drugs are attracting increasing attention from researchers [9].

Exchange protein directly activated by cyclic adenosine monophosphate (cAMP) (Epac) is a recently described cAMP effector molecule that functions in parallel to protein kinase A (PKA) in cAMP signaling. In the heart, cAMP regulates many physiological functions such as contractility, relaxation, and automaticity [10]. Until now, two Epac isoforms have been identified in human cells, Epac1 and Epac2, which are encoded by independent genes and tend to be expressed in different types of cells. Epac1 is mainly found in the heart, kidney, blood vessels, and central nervous system, whereas Epac2 is expressed in the central nervous system and the adrenal glands. Both Epac subtypes increase intracellular cAMP levels in a PKA-independent manner and act on the same direct downstream effector molecules, namely, the small G proteins Rap1 and Rap2 [11, 12]. Many studies that have explored the effects of cAMP signaling on cell death have focused on PKA. However, cAMP signal transduction also occurs via the stimulation of Epac [13, 14]. Previous studies have shown that Epac, which is located on the nuclear membrane of cardiomyocytes, induces sarcoplasmic reticulum calcium leakage to promote cardiac remodeling [15]. In addition, there is evidence that MIRI stimulates cAMP production, activates Epac1, and leads to mitochondrial Ca^2+^ overload, the opening of mitochondrial permeability transition pores (MPTPs), a decline in ATP production, and excessive ROS production, ultimately leading to cardiomyocyte apoptosis [16]. However, relatively little is known about the role of Epac in apoptosis during MIRI.

Vitexin (apigenin-8-C-*β*-D-glucopyranoside, VT) is a flavonoid compound derived from natural products. Previous reports have demonstrated that VT has anti-inflammatory, antihypertensive, antineoplastic, and analgesic effects [17–20]. Our previous studies have shown that VT has protective effects both on MIRI in vivo and hypoxia/reoxygenation- (H/R-) induced injury of cardiomyocytes in vitro [21–25]. However, its underlying mechanisms have not been fully elucidated. The purpose of this study was to elucidate the protective role of VT in inhibiting ischemic myocardial mitochondrial dysfunction and reducing cardiomyocyte apoptosis. In this study, we showed that VT could inhibit the expression of Epac1 and Rap1 during MIRI. In addition, we found that VT could maintain the balance of mitochondrial dynamics by regulating the expression of Drp1 and MFN2 via Epac1-Rap1 signaling, thereby inhibiting mitochondrial-mediated apoptosis.

## 2. Materials and Methods

### 2.1. Animal Preparation

Specific pathogen-free (SPF) male Sprague Dawley (SD) rats (9–11 weeks old, weighing 240–260 g) were purchased from the Experimental Animal Center of Anhui Medical University (license: SCXK (Wan) 2017-001, Hefei, China). All rats were bred and housed in an environment-controlled room (temperature: 22 ± 3°C, humidity: 55% ± 15%, light: 12 h light/12 h dark cycle), with ad libitum access to a standard rodent diet and water. All experimental procedures were conducted in accordance with the guidelines of the National Institutes of Health (NIH) and were compliant with the Institutional Animal Care and Use Committee of Anhui Medical University guidelines (license: SYXK (Wan) 2017-006).

### 2.2. Drugs and Reagents

VT was provided by Qi-xing Medicine and Technology Co., Ltd. (Hefei, China). The 8-CPT-cAMP reagent (an agonist of Epac) was obtained from Abcam (UK). ESI-09 (inhibitor of Epac) was purchased from Selleck Chemicals (USA).

### 2.3. High-Performance Liquid Chromatography (HPLC) and Nuclear Magnetic Resonance (NMR) Spectroscopy Analyses

HPLC (AB Sciex, USA) fingerprint, mass spectra, and NMR (Anasazi, USA) analyses of VT have been shown in our previous study [26].

### 2.4. Langendorff Isolated Rat Heart Perfusion

The Langendorff technique for perfusion of isolated rat hearts has been described in a previous study [27]. All rats were anesthetized with thiopentone (65 mg/kg intraperitoneally (i.p.)); each of the hearts was quickly excised and placed in ice-cold Krebs–Henseleit (K-H) buffer. Every heart was gently pressed to remove residual blood and then mounted on the Langendorff apparatus. The heart was subjected to retrograde perfusion at a constant flow with K-H buffer (aerated with 95% O_2_ and 5% CO_2_) at 37°C and 70 mmHg. A water-filled polyethylene balloon was inserted into the left ventricle (LV) through the left atrium and connected to a pressure sensor via a fluid-filled tube to measure cardiac hemodynamics. Left ventricular end-diastolic pressure (LVEDP) was maintained at 5–10 mmHg by adjusting the volume of the water-filled balloon. Cardiac hemodynamic parameters were recorded using a biological function acquisition system (Chengdu Taimeng Technology Co., Ltd., China) that measured LVEDP, left ventricular systolic pressure (LVSP), heart rate (HR), maximum change rate of left ventricular pressure (±dP/dt_max_), and coronary flow (CF).

Isolated rat hearts were randomly divided into seven groups (*n* = 10 per group): vehicle control, I/R, VT+I/R, I/R+8-CPT, VT+I/R+8-CPT, I/R+ESI-09, and VT+I/R+ESI-09. The drug concentrations used were 10 *μ*M VT, 30 *μ*M 8-CPT, and 1 *μ*M ESI-09. In the control group, the rat hearts received a 90 min constant infusion of KH buffer alone. In the I/R group, the rat hearts were perfused with KH buffer for 30 min, followed by a 30 min cessation of perfusion, and finally 30 min of washout with buffer alone. In the I/R+drug groups, the rat hearts were perfused with KH buffer for 10 min, then perfused with KH buffer containing corresponding drugs for 20 min, followed by cessation of perfusion for 30 min, and finally resumption of KH drug perfusion for 30 min.

### 2.5. Cell Culture and Cardiomyocyte Model of H/R

The rat H9c2 cardiomyocyte cell line was purchased from the Shanghai Cell Bank of the Chinese Academy of Sciences (Shanghai, China). The H9c2 cells were cultured in Dulbecco's modified Eagle's medium/nutrient mixture F-12 (DMEM/F-12) (Biological Industries, Israel) supplemented with 15% fetal bovine serum (FBS) (CellMax, Australia), 1% penicillin (100 U/mL), and 1% streptomycin (100 *μ*g/mL) in an incubator (Thermo Fisher Scientific, USA) at 37°C in a humidified atmosphere of 21% O_2_ and 5% CO_2_.

H9c2 cells were randomly divided into seven groups: control, H/R, VT+H/R, H/R+8-CPT, VT+H/R+8-CPT, H/R+ESI-09, and VT+H/R+ESI-09. The drug concentrations used in these experiments were 10 *μ*M VT, 30 *μ*M 8-CPT, and 1 *μ*M ESI-09. In the control group, H9c2 cells were cultured in normal medium under normal atmospheric conditions (37°C, 21% O_2_, and 5% CO_2_). In the H/R group, the H9c2 cells were cultured with Krebs-Ringer Bicarbonate (KRB) (NaH_2_PO_4_ (0.90 mM), NaCl (98.51 mM), NaHCO_3_ (6.0 mM), KCl (10.0 mM), CaCl_2_ (2.5 mM), MgSO_4_ (1.2 mM), HEPES (20.0 mM), 0.01% BSA, and pH =7.2–7.4) buffer for 5 h in a hypoxic chamber (37°C, 1% O_2_, and 5% CO_2_), after which the KRB buffer was replaced with normal medium and cultured for a further 2 h in a normal atmosphere. In the H/R+different drug groups, the H9c2 cells were incubated with the corresponding drug for 2 h under normal atmospheric conditions. The drug-containing medium was replaced with KRB buffer, followed by culture under hypoxic and reoxygenation conditions as described above.

### 2.6. Determination of Myocardial Infarction

Myocardial infarction in the rats was determined by triphenyltetrazolium chloride (TTC) staining (Beyotime Institute of Biotechnology, China). At the end of reperfusion, the rat hearts were frozen at -20°C for 30 min, sliced into 6–7 sections, and incubated with 1% TTC in phosphate-buffered saline (PBS, pH = 7.4) for 30 min at 37°C in the dark. Sections were photographed with a digital camera, and images were analyzed using ImageJ (National Institutes of Health, USA) software.

### 2.7. Hematoxylin-Eosin (H&E) Staining

Myocardial histopathological damage in the rats was evaluated using H&E staining. At the end of reperfusion, the left ventricular tissues of the rats were fixed in 4% paraformaldehyde for 24 h, and the myocardial specimens were sliced into sections with a thickness of 4 *μ*m after dehydration, clearing, dewaxing, and embedding. The sections were processed with xylene I/II and an ethanol gradient and then stained with hematoxylin for 5 min. The sections were treated with acetic acid and ammonia and then stained with eosin for 2 min. Finally, the sections were dehydrated and sealed. Myocardial tissue sections were examined and photographed using a light microscope (Nikon, Japan).

### 2.8. Terminal Deoxynucleotidyl Transferase-Mediated dUTP Nick-End Labeling (TUNEL) Staining

Myocardial apoptosis was determined using a TUNEL staining kit (Roche, Basel, Switzerland). At the end of reperfusion, rat left ventricular tissue was fixed in 4% paraformaldehyde for 24 h and sliced into sections with a thickness of 4 *μ*m after dehydration, clearing, dewaxing, and embedding. The sections were treated with xylene I/II and an ethanol gradient and then incubated with proteinase K solution for 30 min at 37°C. The sections were incubated with TUNEL staining solution in the dark for 60 min at 37°C and then counterstained in hematoxylin solution for 60 s. Finally, myocardial tissue sections were sealed, examined, and photographed using fluorescence microscopy (Nikon, Japan). The number of TUNEL-positive cells was expressed as a percentage of the total number of cells counted across five random fields of view.

### 2.9. Transmission Electron Microscopy (TEM) of Myocardial Mitochondria

The ultrastructure of myocardial mitochondria was observed and photographed using a TEM (Hitachi, Japan). The preparation of the samples for TEM has been described previously [25].

### 2.10. Mitochondrial Isolation

Mitochondria in cardiomyocytes from myocardial tissue and H9c2 cells were isolated using a Tissue Mitochondrial Isolation Kit and a Cell Mitochondrial Isolation Kit (both Beyotime Institute of Biotechnology, China), respectively. These experimental procedures have been described in our previous study [25].

### 2.11. ROS Assay

The ROS level of H9c2 cells was detected using a Reactive Oxygen Species Assay Kit (Beyotime Institute of Biotechnology, China) according to the manufacturer's instructions. The reagent used in this kit, 2′,7′-dichlorofluorescein diacetate (DCFH-DA), has no intrinsic fluorescence. Exposure to ROS in cells results in the oxidation of DCFH-DA to the fluorescent product DCF, which allows the level of ROS in cells to be measured by detecting the fluorescence intensity of DCF. H9c2 cells were incubated with DCFH-DA for 20 min in an incubator (Thermo Fisher Scientific, USA) at 37°C, and the cells were then washed with PBS. Peroxidation levels were investigated and photographed using a laser confocal microscope (Carl Zeiss, Germany) and quantified using ImageJ software.

### 2.12. Determination of MMP

The MMP in H9c2 cells was determined using an MMP kit with JC-1 (Beyotime Institute of Biotechnology, China). JC-1 is a fluorescent probe that is widely used to detect MMP. When the MMP is high, JC-1 aggregates in the mitochondrial matrix to form polymers (J-aggregates), which produce red fluorescence. At low MMPs, JC-1 cannot aggregate in the mitochondrial matrix and, as a monomer, it produces green fluorescence. Therefore, the degree of mitochondrial depolarization can be measured by the relative proportion of red and green fluorescence. H9c2 cells were incubated with JC-1 solution for 25 min in an incubator at 37°C. After the cells were washed with PBS, the MMP-associated fluorescence was examined and photographed using a laser confocal microscope and quantified using ImageJ software.

### 2.13. Western Blot Analysis

At the end of reperfusion, the left ventricular myocardial tissue from the rat was homogenized prior to lysis for 30 min with radioimmune precipitation (RIPA) lysis buffer containing phenylmethylsulfonyl fluoride (PMSF) on ice. The lysed tissue was centrifuged for 10 min at 12,000 ×g and 4°C. The supernatant was collected, and the total protein concentration was measured using a BCA protein detection kit (Beyotime Institute of Biotechnology, China). The proteins were separated by 12% sodium dodecyl sulfate-polyacrylamide gel electrophoresis (SDS-PAGE) (voltage: 70 V) and then transferred to a polyvinylidene fluoride (PVDF) membrane (pore size: 0.45 *μ*m, Millipore Corp., USA) at 200 mA for 1 h. PVDF membranes were blocked for 1 h at 22 ± 3°C in TBST (Tris-HCl and Tween 20) buffer containing 5% nonfat milk. Membranes were washed three times with TBST buffer, followed by incubation at 4°C overnight with primary antibodies against MFN2 (1 : 1000), Drp1 (1 : 1000), cytochrome c (Cyt c) (1 : 2000), Bax (1 : 1000), Bcl-2 (1 : 1000), cleaved caspase-3/9 (1 : 1000) (all from Abcam, UK), NADPH oxidase 4 (NOX4) (1 : 1000), *β*-actin (1 : 2500), cytochrome c oxidase subunit IV (COX IV) (1 : 2000), or glyceraldehyde-3-phosphate dehydrogenase (GAPDH) (1 : 2500) (all from Cell Signaling Technology, USA). Following this, the membranes were washed three times with TBST and incubated at room temperature for 1 h with an appropriate horseradish peroxidase-conjugated IgG secondary antibody. Some of the antibodies, such as *β*-actin, COX IV, and GAPDH, were used as internal standards. The immunoreactive bands were visualized using an enhanced chemiluminescence kit (Thermo Fisher Scientific, USA) and a chemiluminescence imaging system (Bioshine, China). Finally, the density of protein immune bands was quantified using ImageJ software.

### 2.14. Statistical Analyses

The data are represented as mean ± SEM. Significant differences in data were assessed using one-way analysis of variance (ANOVA) and Student's *t*-test. The data were analyzed using GraphPad Prism 6.0 (USA), and a *P* < 0.05 was considered statistically significant.

## 3. Results

### 3.1. VT Alleviates Cardiac Dysfunction in Isolated Rat Hearts Subjected to I/R Injury

**Figure 1:**
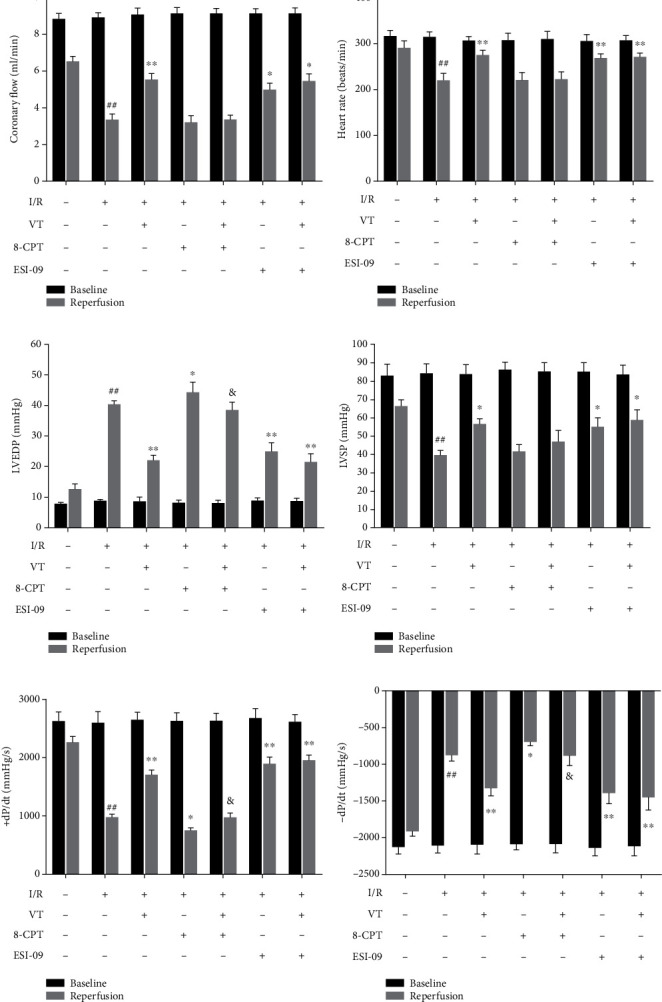
Vitexin alleviates cardiac dysfunction in isolated rat hearts subjected to I/R injury. (a) Coronary flow (CF). (b) Heart rate (HR). (c) Left ventricular end-diastolic pressure (LVEDP). (d) Left ventricular systolic pressure (LVSP). (e) +dP/dt_max_. (f) -dP/dt_max_. The data represent the mean ± SEM (*n* = 6). ^##^*P* < 0.01 vs. the control group; ^∗^*P* < 0.05 and ^∗∗^*P* < 0.01 vs. the I/R group; ^&^*P* < 0.05 vs. the I/R+8-CPT group.

Hemodynamic parameters are important indicators of cardiac function. We analyzed the changes in these parameters in isolated rat hearts using a biological function acquisition system. Our results showed that there was no significant difference in the baseline values of cardiac function parameters between the groups during the perfusion equilibrium phase. At the end of reperfusion, CF (Figure 1(a)), HR (Figure 1(b)), LVSP (Figure 1(d)), and ±dP/dt_max_ (Figures 1(e) and 1(f)) considerably reduced, whereas LVEDP significantly increased in the I/R group compared to that in the control group (Figure 1(c)). Either the administration of VT or ESI-09 alone or in combination significantly alleviated the reduction of cardiac function exhibited by the I/R group.

However, 8-CPT, a chemical chaperone that was reported to agitate Epac signal transduction, aggravated cardiac dysfunction compared to that in the I/R group. In the presence of VT, there was no significant difference in CF (Figure 1(a)), HR (Figure 1(b)), and LVSP (Figure 1(d)), whereas LVEDP significantly decreased (Figure 1(c)), and ±dP/dt_max_ significantly increased compared to that in the I/R+8-CPT group (Figures 1(e) and 1(f)).

### 3.2. VT Reduces Myocardial Infarct Size and Pathological Damage in Isolated Rat Hearts Subjected to I/R Injury

**Figure 2:**
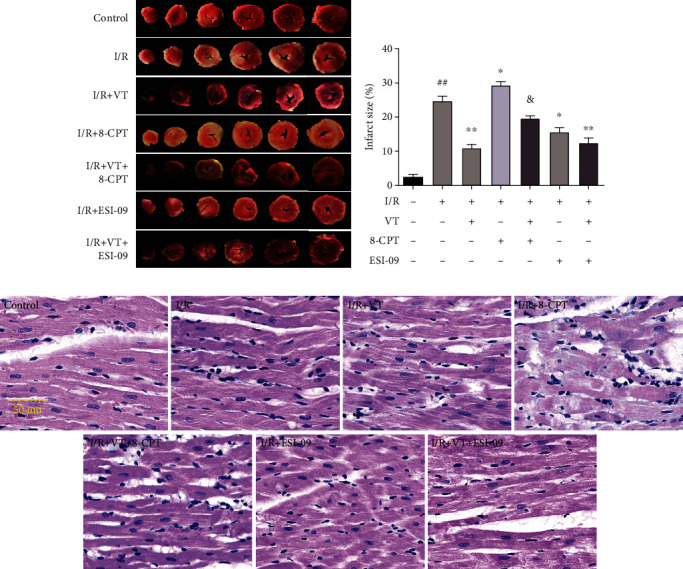
Vitexin reduces myocardial infarct size and pathological damage in isolated rat hearts subjected to I/R injury. (a) Representative images of cardiac TTC staining. (b) Quantitative graph of myocardial infarct size. (c) Representative images of cardiac H&E staining. The data represent the mean ± SEM (*n* = 6). ^##^*P* < 0.01 vs. the control group; ^∗^*P* < 0.05 and ^∗∗^*P* < 0.01 vs. the I/R group; ^&^*P* < 0.05 vs. the I/R+8-CPT group.

The myocardial infarct size was assessed using TTC staining. Our results showed that the myocardial infarct size significantly increased in MIRI compared to that in the control group. This severe I/R-induced increase in myocardial infarct size was significantly reduced with VT or ESI-09 administration alone or in combination. Conversely, 8-CPT increased the myocardial infarct size, while VT treatment reversed these changes (Figures 2(a) and 2(b)). In addition, myocardial histopathological damage detected by H&E staining exhibited matching changes. The H&E staining showed that, in the control group, the cardiomyocytes were normal and arranged in order. In comparison, cardiomyocytes in the I/R group were disordered and the nuclei were shrunken and more deeply stained. Interestingly, compared to the I/R group, the cardiomyocytes in the I/R+VT, I/R+ESI-09, and I/R+VT+ESI-09 groups were arranged in an orderly manner, and the nuclear staining was normal, whereas the cardiomyocyte pathological damage was more serious in the I/R+8-CPT group. This damage was alleviated in the I/R+VT+8-CPT group (Figure 2(c)).

### 3.3. VT Reduces Cardiomyocyte Apoptosis in Isolated Rat Hearts Subjected to I/R Injury

**Figure 3:**
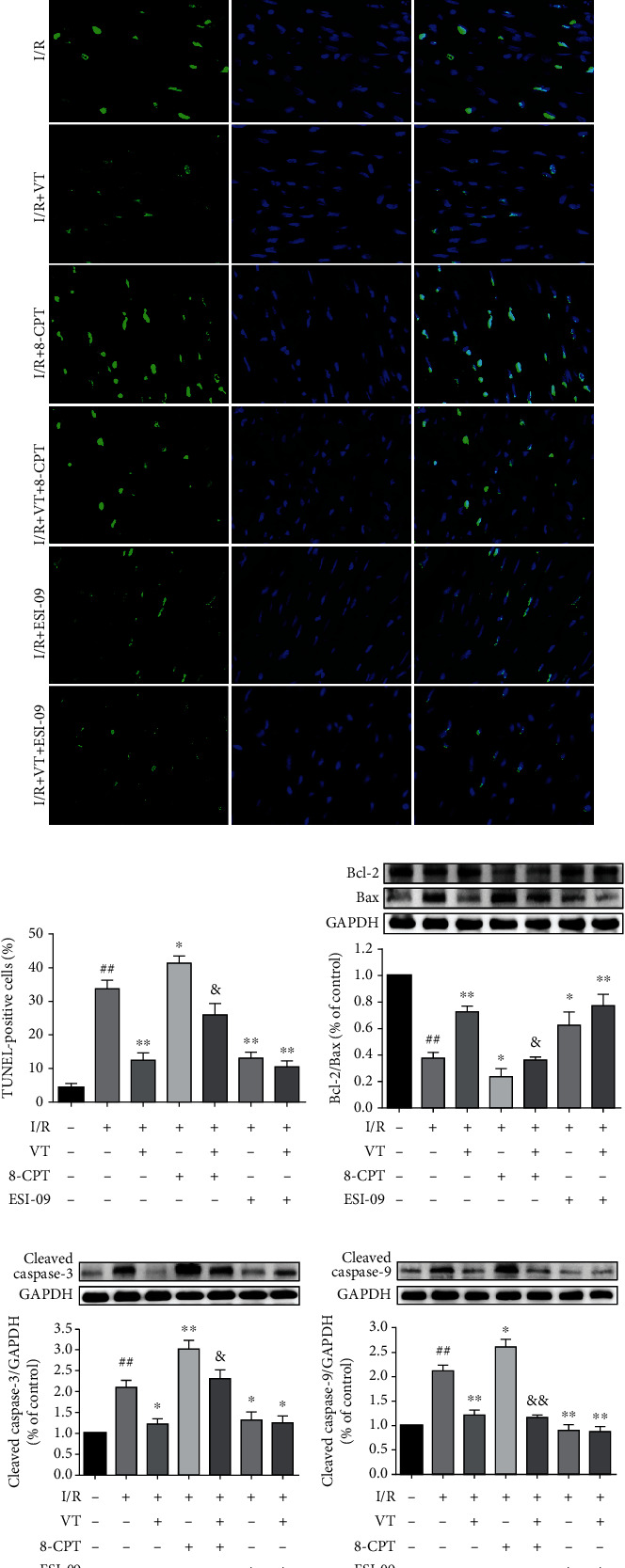
Vitexin reduces cardiomyocyte apoptosis in isolated rat hearts subjected to I/R injury. (a) Representative fluorescence images of myocardial TUNEL staining. (b) Quantitative graph of TUNEL-positive cells. Western blot analyses showing the expression of (c) Bcl-2 and Bax, (d) cleaved caspase-3, and (e) cleaved caspase-9. The data represent the mean ± SEM (*n* = 4). ^##^*P* < 0.01 vs. the control group; ^∗^*P* < 0.05 and ^∗∗^*P* < 0.01 vs. the I/R group; ^&^*P* < 0.05 vs. the I/R+8-CPT group.

TUNEL staining showed that the number of apoptotic positive cells was higher in the I/R group compared to that in the control group. The number of apoptotic positive cells was significantly reduced by VT, by ESI-09, or by combination treatment compared to that in the I/R group. In contrast, 8-CPT treatment increased the number of apoptotic positive cells, whereas in the presence of VT, this number was lowered (Figures 3(a) and 3(b)). In addition, the expression of the apoptosis-related proteins Bax, Bcl-2, cleaved caspase-3, and cleaved caspase-9 was analyzed using western blotting. Our results showed that, compared to that in the control group, the ratio of Bcl-2/Bax was significantly reduced (Figure 3(c)), and the expression of cleaved caspase-3 and cleaved caspase-9 was significantly increased in the I/R group (Figures 3(d) and 3(e)). Compared to that in the I/R group, the ratio of Bcl-2/Bax significantly increased (Figure 3(c)), and the expression of cleaved caspase-3 and cleaved caspase-9 was downregulated in the I/R+VT, I/R+ESI-09, and I/R+VT+ESI-09 groups, whereas the ratio of Bcl-2/Bax was significantly reduced, and the expression of cleaved caspase-3 and cleaved caspase-9 significantly increased in the I/R+8-CPT group. The proapoptotic changes observed in the I/R+8-CPT group were diminished by the inclusion of VT (Figures 3(d) and 3(e)).

### 3.4. VT Reduces ROS Levels in H9c2 Cells Subjected to H/R Injury

**Figure 4:**
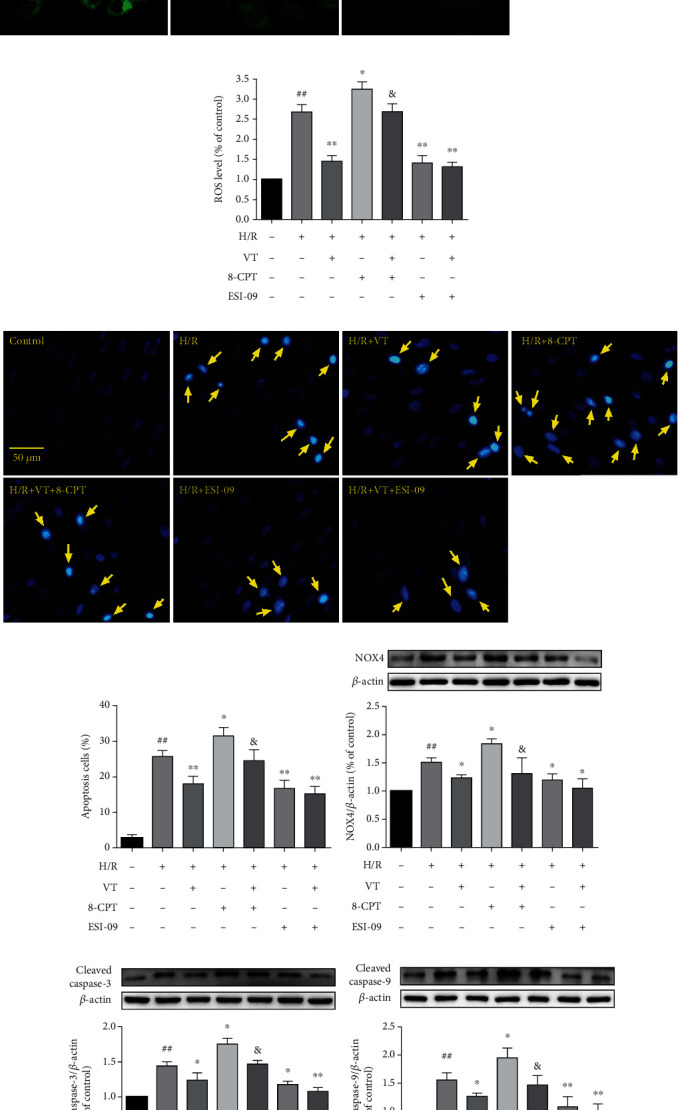
Vitexin reduces ROS levels and apoptosis in H9c2 cells subjected to H/R injury. (a) Representative fluorescence images of ROS. (b) Quantitative graph of ROS levels. (c) Representative fluorescence images of Hoechst staining. (d) Quantitative graph of apoptotic cells. Western blot analyses showing the expression of (e) NOX4, (f) cleaved caspase-3, and (g) cleaved caspase-9. The data represent the mean ± SEM (*n* = 4). ^##^*P* < 0.01 vs. the control group; ^∗^*P* < 0.05 and ^∗∗^*P* < 0.01 vs. the H/R group; ^&^*P* < 0.05 vs. the H/R+8-CPT group.

Our results showed that, compared to that in the control group, ROS levels in H9c2 cells significantly increased in the H/R group. Compared to that in the H/R group, the administration of VT or ESI-09 alone or in combination significantly reduced the ROS levels. Conversely, 8-CPT treatment increased ROS levels, whereas the addition of VT to 8-CPT treatment lowered the ROS levels (Figures 4(a) and 4(b)). Furthermore, western blotting analyses showed that the expression of NOX4 exhibited a similar trend to ROS levels, with a significant increase in the H/R group compared to the control group, which was significantly reduced by VT, by ESI-09, or by combination treatment (Figure 4(e)). In contrast, 8-CPT treatment increased the expression of NOX4 compared to that in the H/R group, whereas the addition of VT significantly decreased NOX4 expression (Figure 4(e)).

### 3.5. VT Reduces Apoptosis in H9c2 Cells Subjected to H/R Injury

The results of Hoechst staining showed that there were a higher number of apoptotic cells in the H/R group than in the control group and that this increase in apoptotic cells was reduced with VT, ESI-09, or by combination treatment (Figures 4(c) and 4(d)). As expected, 8-CPT treatment increased the number of apoptotic cells. However, this effect was abrogated in the presence of VT (Figures 4(c) and 4(d)). These data matched the results from the isolated rat heart cardiomyocytes. In keeping with these findings, western blotting showed that the expression of cleaved caspase-3 and cleaved caspase-9 in the H/R group was significantly higher than that in the control group, that it was significantly reduced by VT, ESI-09, or combination treatment, and that the increase induced by 8-CPT was reduced by the inclusion of VT (Figures 4(f) and 4(g)).

### 3.6. VT Alleviates Mitochondrial Structural Damage in Isolated Rat Hearts Subjected to I/R Injury

**Figure 5:**
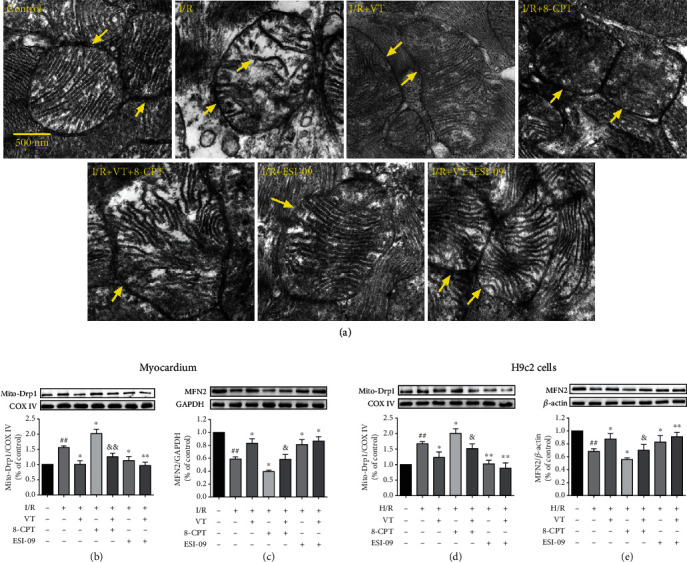
Vitexin improves mitochondrial structures and reverses the MIRI-induced decrease and increase in MFN2 and Drp1 expression, respectively. (a) Representative TEM images of myocardial mitochondria. Western blot analyses showing the expression of (b) Drp1 in myocardial mitochondria, (c) MFN2 in myocardium, (d) Drp1 in mitochondria of H9c2 cells, and (e) MFN2 in H9c2 cells. The data represent the mean ± SEM (*n* = 4). ^##^*P* < 0.01 vs. the control group; ^∗^*P* < 0.05 and ^∗∗^*P* < 0.01 vs. the I/R group or the H/R group; ^&^*P* < 0.05 vs. the I/R+8-CPT group or the H/R+8-CPT group.

Representative transmission electron micrographs of the cardiac mitochondria showed that, compared to that in the control group, the outer mitochondrial membrane was ruptured, and the mitochondrial cristae were broken and even vacuolated in the I/R group (Figure 5(a)). Interestingly, compared to that in the I/R group, the administration of VT or ESI-09 alone or in combination had a protective effect, with evidence of more complete outer mitochondrial membranes and cristae. Conversely, 8-CPT caused severe damage to the mitochondrial structures compared to that in the I/R group; however, VT treatment alleviated this structural damage (Figure 5(a)).

### 3.7. VT Affects the Expression of Mitochondrial Fission and Fusion Proteins

Western blot analyses of proteins from isolated rat hearts showed that in the I/R group, the expression of Drp1 significantly increased compared to that in the control group (Figure 5(b)), whereas the expression of MFN2 significantly decreased. Compared to that in the I/R group (Figure 5(c)), the expression of Drp1 significantly reduced and that of MFN2 significantly increased with VT, ESI-09, or combination treatment, whereas in the presence of 8-CPT, the expression of Drp1 increased and that of MFN2 decreased, with the addition of VT abrogating these changes (Figures 5(b) and 5(c)). Furthermore, western blot analyses of H9c2 cells treated in a similar fashion supported these results (Figures 5(d) and 5(e)).

### 3.8. VT Increases the Mitochondrial Membrane Potential in H9c2 Cells Subjected to H/R Injury

**Figure 6:**
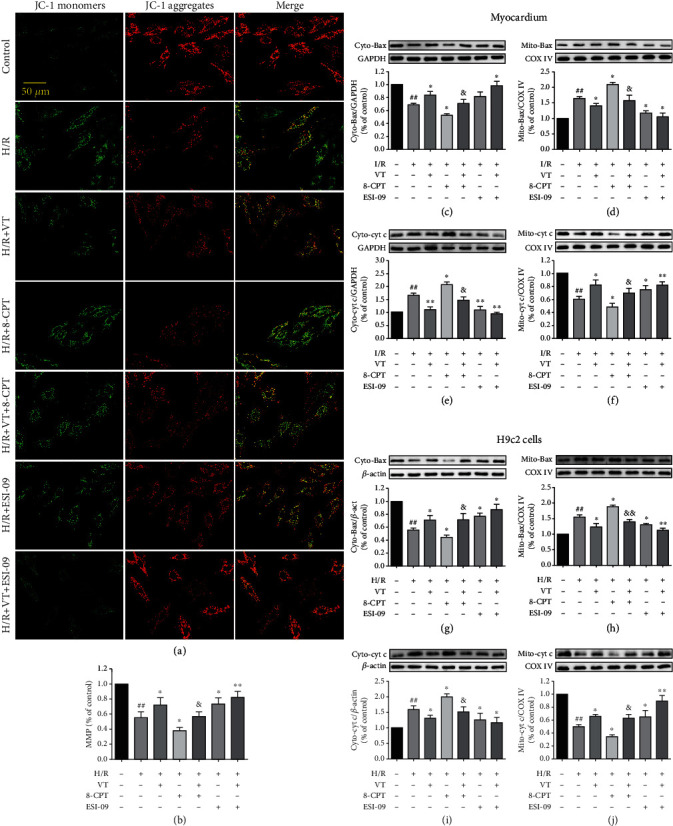
Vitexin affects MMP, Cyt c release, and Bax recruitment in mitochondria. (a) Representative fluorescence images of JC-1 staining in H9c2 cells. (b) Quantitative graph of MMP. Western blot analyses showing the expression of (c) cytoplasmic and (d) mitochondrial Bax in myocardium, (e) cytoplasmic and (f) mitochondrial Cyt c in myocardium, (g) cytoplasmic and (h) mitochondrial Bax in H9c2 cells, and (i) cytoplasmic and (j) mitochondrial Cyt c in H9c2 cells. The data represent the mean ± SEM (*n* = 4). ^##^*P* < 0.01 vs. the control group; ^∗^*P* < 0.05 and ^∗∗^*P* < 0.01 vs. the I/R group or the H/R group; ^&^*P* < 0.05 vs. the I/R+8-CPT group or the H/R+8-CPT group.

JC-1 staining showed that MMP significantly decreased in the H/R compared to that in the control group (Figures 6(a) and 6(b)). Interestingly, compared to that in the H/R group, the MMP significantly increased with VT, ESI-09, or with combination treatment, whereas in the presence of 8-CPT, the MMP significantly decreased. Compared with the H/R+8-CPT group, VT treatment significantly increased the MMP in the H/R+VT+8-CPT group (Figures 6(a) and 6(b)).

### 3.9. VT Reduces Cyt c Release and Bax Recruitment in Mitochondria

Western blotting of isolated rat hearts showed that in the I/R compared to that in the control group, the levels of Bax and Cyt c in the cytoplasm significantly decreased and increased (Figures 6(c) and 6(e)), respectively, whereas an inverse pattern of expression was observed for these proteins in the mitochondria (Figures 6(d) and 6(f)). Both cytoplasmic and mitochondrial patterns of expression for Bax and Cyt c were reversed by VT, ESI-09, or with combination treatment, whereas in the presence of 8-CPT, the I/R-induced patterns of expression were exacerbated. In the I/R+VT+8-CPT group, Bax levels increased and Cyt c levels decreased in the cytoplasm (Figures 6(c) and 6(e)), whereas Bax levels decreased and Cyt c levels increased in the mitochondria (Figures 6(d) and 6(f)). Western blot analyses of parallel experiments on H9c2 cells exhibited similar trends, providing further support for these results (Figures 6(g) and 6(j)).

### 3.10. VT Downregulates Epac1 and Rap1 Protein Expression

**Figure 7:**
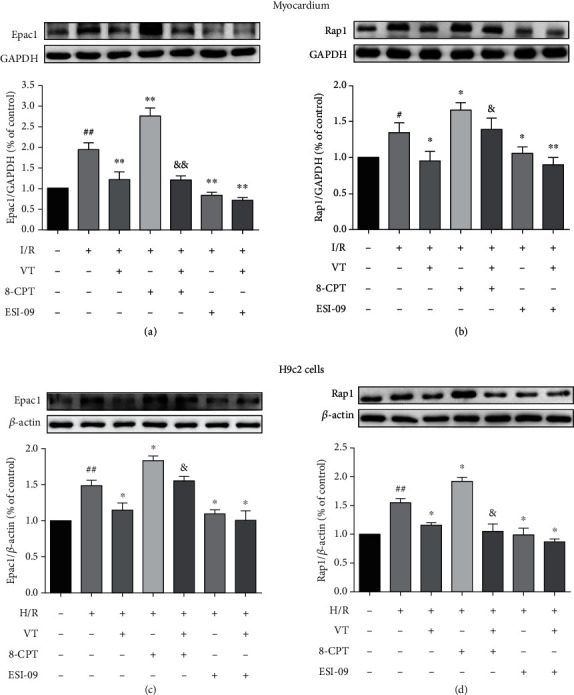
Vitexin downregulates Epac1 and Rap1 protein expression. Western blot analyses showing the expression of (a) Epac1 and (b) Rap1 in myocardium, and (c) Epac1 and (d) Rap1 in H9c2 cells. The data represent the mean ± SEM (*n* = 4). ^##^*P* < 0.01 vs. the control group; ^∗^*P* < 0.05 and ^∗∗^*P* < 0.01 vs. the I/R group or the H/R group; ^&^*P* < 0.05 vs. the I/R+8-CPT group or the H/R+8-CPT group.

Western blotting of isolated rat hearts showed that, compared to that in the control group, the expression of both Epac1 and Rap1 significantly increased in the I/R group (Figures 7(a) and 7(b)). Interestingly, compared to the I/R group, the expression of both proteins was significantly reduced by VT or ESI-09 alone, as well as the combination of these drugs, whereas 8-CPT significantly increased the expression of these proteins in the I/R+8-CPT group. Interestingly, VT treatment significantly reduced the expression of Epac1 and Rap1 compared with that in the I/R+8-CPT group (Figures 7(a) and 7(b)). These results are supported by the outcomes of experiments performed with H9c2 cells (Figures 7(c) and 7(d)).

## 4. Discussion

The restoration of coronary blood flow is the most effective intervention to minimize myocardial ischemic injury in order to improve survival. Clinical interventions that restore the blood supply, such as coronary thrombolytic therapy and percutaneous coronary intervention, can alleviate the cardiomyocyte metabolic dysfunction caused by ischemia. Paradoxically, the reperfusion process can also cause additional myocardial damage. This study provides evidence of the core role of mitochondrial function in the pathogenesis of MIRI. In addition, we found that VT could alleviate MIRI-induced mitochondrial dysfunction via the Epac1-Rap1 signaling pathway, thereby mitigating MIRI. These findings have substantiated the sufficiency of maintaining the balance of mitochondrial dynamics to mitigate MIRI and the necessity of VT to inhibit Epac1 activation.

cAMP is an important signaling molecule that regulates the physiological activity and metabolism of cells [28, 29]. In multicellular eukaryotes, cAMP functions by binding two commonly expressed intracellular cAMP receptors, the classic cAMP-dependent receptor PKA and the more recently discovered Epac [30, 31]. A recent study by Fazal et al. reported that Epac1 is involved in mitochondrial pathway-mediated cardiomyocyte apoptosis. The authors showed that knockout of the *Epac1* gene or pharmacological intervention prevented experimental MIRI and reduced both the infarct size and cardiomyocyte apoptosis [16]. In addition, Wang et al. found that H/R treatment significantly reduced the expression of Epac1 protein in bone marrow mesenchymal stem cells. However, treatment of these cells with curcumin significantly increased the level of Epac1 protein after H/R, thereby maintaining mitochondrial function and mitigating the effects of H/R [32]. Our study showed that the expression of Epac1 and Rap1 was significantly upregulated after I/R. In addition, I/R induced myocardial infarction and left ventricular dysfunction. To further verify the role of Epac1 in MIRI, we used the Epac1 agonist, 8-CPT. Our results showed that with 8-CPT pretreatment, the expression of Epac1 and Rap1 was upregulated and myocardial damage was further aggravated. In contrast, pretreatment with VT or the Epac1 inhibitor ESI-09 significantly suppressed the expression of Epac1 and Rap1, reduced myocardial infarction, and alleviated left ventricular and mitochondrial dysfunction. Together, these results indicate that the role of VT in mitigating MIRI-associated damage involves the Epac1-Rap1 signaling pathway. In addition, the present study suggests that the upregulation of Epac1 expression may be one of the mechanisms underpinning MIRI. During cardiac ischemia-reperfusion in rats, the expression of Epac1 in the heart was upregulated, activating the downstream protein Rap1 to initiate a signal transduction cascade that resulted in the inhibition of mitochondrial fusion, promotion of mitochondrial fission, disruption of mitochondrial homeostasis and function, and activation of mitochondrial pathway-mediated cardiomyocyte apoptosis. Our findings suggest that inhibition of Epac1 expression could alleviate MIRI and that the Epac1-Rap1 signaling pathway may be an effective target for the treatment of ischemic myocardial injury.

Many studies have shown that apoptosis plays a crucial role in the progression and development of cardiovascular diseases [33], and interventions that inhibit apoptosis could effectively alleviate MIRI. The members of the cysteinyl aspartate-specific proteinase (caspase) family are important participants in apoptosis, of which caspase-9 is the initiator of the mitochondrial endogenous apoptosis pathway, and caspase-3 is the ultimate mediator of apoptosis [34]. I/R induces mitochondrial damage, resulting in reduced oxidative phosphorylation and the opening of MPTPs [35]. At the same time, Bcl-2 activity is inhibited and Bax is recruited to the mitochondria. Furthermore, the mitochondria release proapoptotic factors such as Cyt c to activate caspase-9, thereby initiating programmed cell death [36]. A study by Xie et al. showed that VT was capable of alleviating ER stress-activated apoptosis and the related inflammation in rat chondrocytes [37]. Furthermore, Min et al. showed that VT protected against hypoxic-ischemic injury by inhibiting Ca^2+^/calmodulin-dependent protein kinase II and apoptosis signaling in the neonatal mouse brain [38]. Our previous study showed that VT exerted cardioprotective effects on chronic MIRI in rats by inhibiting myocardial apoptosis and lipid peroxidation [26]. Together, these studies showed that VT could effectively inhibit apoptosis. Interestingly, VT can also induce apoptosis in cancer cells [39, 40]. In this study, we showed that VT inhibited the release of Cyt c and the recruitment of Bax in mitochondria during MIRI via the Epac1-Rap1 signaling pathway. Furthermore, VT inhibited the expression of cleaved caspase-3/9 and increased the Bcl-2/Bax ratio during MIRI. These data indicate that VT inhibits the activation of the mitochondrial-mediated apoptotic pathway during MIRI via Epac1-Rap1 signaling.

Changes in mitochondrial structure and function are important signs of mitochondrial dysfunction [41, 42]. This study showed that VT inhibited I/R-induced rupture of both the outer mitochondrial membrane and the cristae, as well as vacuolation. Mitochondria are the main sites of ROS production, which occurs primarily during mitochondrial oxygen metabolism. Deregulated levels of these critical signaling molecules are largely responsible for mitochondrial dysfunction [43], and oxidative stress caused by excessive ROS is an early feature of mitochondrial apoptosis [44]. During I/R, excessive ROS causes direct disruption of mitochondrial membrane permeability. MMP is a key indicator of mitochondrial function, and its homeostasis is highly dependent on the integrity of the mitochondrial membrane. Excessive ROS can stimulate the opening of MPTPs, leading to a decrease in MMP and the release of proapoptotic factors such as Cyt c [45]. In general, mitochondria are the key organelles responsible for cellular energy production, and the disruption of MMP ultimately reduces ATP production [46]. Our data showed that VT reduced ROS levels and increased the MMP in H9c2 cells subjected to H/R injury. Moreover, VT reduced the release of Cyt c, the recruitment of Bax in mitochondria, and the expression of cleaved caspase-3/9 in H9c2 cells subjected to H/R injury. NADPH oxidase 4 (NOX4) is the main source of ROS in the heart [47]. Considering the important role of ROS in mitochondrial function, we evaluated the expression of NOX4. Our results showed that VT inhibited the expression of NOX4 in H9c2 cells subjected to H/R injury, suggesting that inhibition of NOX4 may be a therapeutic strategy to alleviate MIRI.

Mitochondria are highly dynamic organelles that maintain their normal function through the balance of mitochondrial dynamics (fusion/fission) [48]. Disruption of this balance plays an important role in myocardial I/R injury [49, 50]. Related studies have confirmed that I/R inhibits the expression of mitochondrial fusion-related proteins, including MFN1, MFN2, and OPA1, and increases cardiomyocyte apoptosis [51, 52]. Zhao et al. indicated that cardiomyocytes from *Mfn2* knockout mice exhibited a substantial loss of MMP and a significant reduction in survival after I/R, suggesting that downregulation of MFN2 aggravates mitochondrial damage and cardiomyocyte apoptosis [53]. In addition, it has been reported that inhibition of MFN2 may reduce the MMP and increase the release of Cyt c [54]. Our results are consistent with these observations. The above studies show that promoting mitochondrial fusion has cardioprotective effects during I/R. However, some studies have reported findings that are in direct opposition to these. For example, Hall et al. reported that when mouse hearts lacking both MFN1 and MFN2 were subjected to I/R, there was a reduction in the myocardial infarct size [55]. This may be related to the nonfusion roles of these fusion proteins. In general, excessive mitochondrial fission plays a key role in promoting myocardial apoptosis during I/R. Upregulation of MFF is capable of increasing mitochondrial ROS production and reducing both MMP and oxygen consumption [56]. Parra et al. showed that the upregulation of mitochondrial fission proteins such as Drp1, Fis1, and MFF led to increased mitochondrial fission and apoptosis in cardiomyocytes subjected to I/R [54]. I/R also induces Drp1 recruitment from the cytoplasm to the mitochondria [57]. Inhibition of Drp1 overexpression reduces myocardial infarct size and cardiomyocyte apoptosis and alleviates left ventricular dysfunction [58, 59]. Our research is consistent with these findings as well. The present study showed that VT inhibited the MIRI-induced decrease and increase in MFN2 and Drp1 expression, respectively, thereby maintaining mitochondrial dynamic balance, as indicated by a decrease in ROS levels, an increase in MMP, and the integrity of mitochondrial structures. These results indicate that VT may reduce MIRI-induced mitochondrial dysfunction by maintaining mitochondrial dynamic balance.

In summary, we established ex vivo I/R models and in vitro H/R models as proxies for investigating MIRI. We found that the upregulation of Epac1 was associated with mitochondrial dysfunction and promoted mitochondrial-mediated apoptosis. Interestingly, we demonstrated that VT inhibits Epac1 expression during I/R. Furthermore, we observed that VT or an Epac inhibitor alleviated mitochondrial dysfunction by increasing MMP, reducing ROS production, increasing MFN2 expression, and inhibiting Drp1 expression. The present study showed that VT treatment may alleviate MIRI, at least in part, by mitigating MIRI-induced mitochondrial dysfunction through the Epac1-Rap1 signaling pathway, thereby inhibiting activation of mitochondrial-mediated apoptosis. Additionally, our results provide new insights into the protective mechanisms underpinning the effect of VT on MIRI and basis for VT and Epac in the clinical treatment of ischemic heart disease. Nevertheless, our study has some limitations. We did not explore the specific regulatory mechanism of Epac during I/R, and further studies are required to determine the therapeutic potential of VT via Epac1.

## Data Availability

The underlying data supporting the results of our study in this manuscript are stored in the archives of Anhui Medical University, and the backup is available in the Department of Pharmacology.

## Additional Points

*Highlights*. (i) Vitexin has protective effects against myocardial ischemia/reperfusion injury. (ii) Vitexin alleviated mitochondrial dysfunction induced by myocardial ischemia/reperfusion injury. (iii) Vitexin reversed the MIRI-induced decrease and increase in MFN2 and Drp1 expression, respectively.

## References

[1] Hausenloy D. J., Yellon D. M. (2013). Myocardial ischemia-reperfusion injury: a neglected therapeutic target. *The Journal of Clinical Investigation*.

[2] Hausenloy D. J., Yellon D. M. (2016). Ischaemic conditioning and reperfusion injury. *Nature Reviews Cardiology*.

[3] Yellon D. M., Hausenloy D. J. (2007). Myocardial reperfusion injury. *New England Journal of Medicine*.

[4] Frohlich G. M., Meier P., White S. K., Yellon D. M., Hausenloy D. J. (2013). Myocardial reperfusion injury: looking beyond primary PCI. *European Heart Journal*.

[5] Dong G., Chen T., Ren X. (2016). Rg1 prevents myocardial hypoxia/reoxygenation injury by regulating mitochondrial dynamics imbalance via modulation of glutamate dehydrogenase and mitofusin 2. *Mitochondrion*.

[6] Madungwe N. B., Zilberstein N. F., Feng Y., Bopassa J. C. (2016). Critical role of mitochondrial ROS is dependent on their site of production on the electron transport chain in ischemic heart. *American Journal of Cardiovascular Disease*.

[7] Matoba T., Koga J. I., Nakano K., Egashira K., Tsutsui H. (2017). Nanoparticle-mediated drug delivery system for atherosclerotic cardiovascular disease. *Journal of Cardiology*.

[8] Li H. W., Xiao F. Y. (2020). Effect of hydrogen sulfide on cardiomyocyte apoptosis in rats with myocardial ischemia-reperfusion injury via the JNK signaling pathway. *European Review for Medical and Pharmacological Sciences*.

[9] Gu L. L., Shen Z. L., Li Y. L., Bao Y. Q., Lu H. (2018). Oxymatrine causes hepatotoxicity by promoting the phosphorylation of JNK and induction of endoplasmic reticulum stress mediated by ROS in LO2 cells. *Molecules and Cells*.

[10] El-Armouche A., Eschenhagen T. (2009). Beta-adrenergic stimulation and myocardial function in the failing heart. *Heart Failure Reviews*.

[11] de Rooij J., Zwartkruis F. J., Verheijen M. H. (1998). Epac is a Rap1 guanine-nucleotide-exchange factor directly activated by cyclic AMP. *Nature*.

[12] Kawasaki H., Springett G. M., Mochizuki N. (1998). A family of cAMP-binding proteins that directly activate Rap 1. *Science*.

[13] Insel P. A., Zhang L., Murray F., Yokouchi H., Zambon A. C. (2012). Cyclic AMP is both a pro-apoptotic and anti-apoptotic second messenger. *Acta Physiologica (Oxford, England)*.

[14] Yu Q. J., Si R., Zhou N. (2008). Insulin inhibits beta-adrenergic action in ischemic/reperfused heart: a novel mechanism of insulin in cardioprotection. *Apoptosis*.

[15] Laurent A. C., Bisserier M., Lucas A. (2015). Exchange protein directly activated by cAMP 1 promotes autophagy during cardiomyocyte hypertrophy. *Cardiovascular Research*.

[16] Fazal L., Laudette M., Paula-Gomes S. (2017). Multifunctional mitochondrial Epac 1 controls myocardial cell death. *Circulation Research*.

[17] Prabhakar M. C., Bano H., Kumar I., Shamsi M. A., Khan M. S. (1981). Pharmacological investigations on vitexin. *Planta Medica*.

[18] Lee C. Y., Chien Y. S., Chiu T. H. (2012). Apoptosis triggered by vitexin in U937 human leukemia cells via a mitochondrial signaling pathway. *Oncology Reports*.

[19] Demir Ozkay U., Can O. D. (2013). Anti-nociceptive effect of vitexin mediated by the opioid system in mice. *Pharmacology, Biochemistry, and Behavior*.

[20] Yang L., Yang Z. M., Zhang N., Tian Z., Liu S. B., Zhao M. G. (2014). Neuroprotective effects of vitexin by inhibition of NMDA receptors in primary cultures of mouse cerebral cortical neurons. *Molecular and Cellular Biochemistry*.

[21] Dong L. Y., Chen Z. W., Guo Y., Cheng X. P., Shao X. (2008). Mechanisms of vitexin preconditioning effects on cultured neonatal rat cardiomyocytes with anoxia and reoxygenation. *The American Journal of Chinese Medicine*.

[22] Dong L., Fan Y., Shao X., Chen Z. (2011). Vitexin protects against myocardial ischemia/reperfusion injury in Langendorff-perfused rat hearts by attenuating inflammatory response and apoptosis. *Food and Chemical Toxicology*.

[23] Dong L. Y., Li S., Zhen Y. L., Wang Y. N., Shao X., Luo Z. G. (2013). Cardioprotection of vitexin on myocardial ischemia/reperfusion injury in rat via regulating inflammatory cytokines and MAPK pathway. *The American Journal of Chinese Medicine*.

[24] Che X., Wang X., Zhang J. (2016). Vitexin exerts cardioprotective effect on chronic myocardial ischemia/reperfusion injury in rats via inhibiting myocardial apoptosis and lipid peroxidation. *American Journal of Translational Research*.

[25] Xue W., Wang X., Tang H. (2020). Vitexin attenuates myocardial ischemia/reperfusion injury in rats by regulating mitochondrial dysfunction induced by mitochondrial dynamics imbalance. *Biomedicine & Pharmacotherapy*.

[26] Wang Y., Zhen Y., Wu X. (2015). Vitexin protects brain against ischemia/reperfusion injury _via_ modulating mitogen-activated protein kinase and apoptosis signaling in mice. *Phytomedicine*.

[27] Jeddi S., Khalifi S., Ghanbari M., Bageripour F., Ghasemi A. (2016). Effects of nitrate intake on myocardial ischemia-reperfusion injury in diabetic rats. *Arquivos Brasileiros de Cardiologia*.

[28] Lefkimmiatis K., Zaccolo M. (2014). cAMP signaling in subcellular compartments. *Pharmacology & Therapeutics*.

[29] Di Benedetto G., Pendin D., Greotti E., Pizzo P., Pozzan T. (2014). Ca2+ and cAMP cross-talk in mitochondria. *The Journal of Physiology*.

[30] Cheng X., Ji Z., Tsalkova T., Mei F. (2008). Epac and PKA: a tale of two intracellular cAMP receptors. *Acta Biochimica et Biophysica Sinica*.

[31] Borland G., Smith B. O., Yarwood S. J. (2009). EPAC proteins transduce diverse cellular actions of cAMP. *British Journal of Pharmacology*.

[32] Wang X., Zhang Y., Yang Y. (2019). Curcumin pretreatment protects against hypoxia/reoxgenation injury via improvement of mitochondrial function, destabilization of HIF-1*α* and activation of Epac1-Akt pathway in rat bone marrow mesenchymal stem cells. *Biomedicine & Pharmacotherapy*.

[33] Jing S. H., Yu B., Qiao H. (2019). Correlation between endothelial cell apoptosis and SIRT3 gene expression in atherosclerosis rats. *European Review for Medical and Pharmacological Sciences*.

[34] Ni H. M., McGill M. R., Chao X., Woolbright B. L., Jaeschke H., Ding W. X. (2016). Caspase Inhibition Prevents Tumor Necrosis Factor-*α*-Induced Apoptosis and Promotes Necrotic Cell Death in Mouse Hepatocytes _in Vivo_ and _in Vitro_. *The American Journal of Pathology*.

[35] Li C., Jackson R. M. (2002). Reactive species mechanisms of cellular hypoxia-reoxygenation injury. *American Journal of Physiology. Cell Physiology*.

[36] Basalay M. V., Davidson S. M., Gourine A. V., Yellon D. M. (2018). Neural mechanisms in remote ischaemic conditioning in the heart and brain: mechanistic and translational aspects. *Basic Research in Cardiology*.

[37] Xie C. L., Li J. L., Xue E. X. (2018). Vitexin alleviates ER-stress-activated apoptosis and the related inflammation in chondrocytes and inhibits the degeneration of cartilage in rats. *Food & Function*.

[38] Min J. W., Kong W. L., Han S. (2017). Vitexin protects against hypoxic-ischemic injury via inhibiting Ca2+/calmodulin-dependent protein kinase II and apoptosis signaling in the neonatal mouse brain. *Oncotarget*.

[39] Bhardwaj M., Cho H. J., Paul S. (2018). Vitexin induces apoptosis by suppressing autophagy in multi-drug resistant colorectal cancer cells. *Oncotarget*.

[40] Liu X., Jiang Q., Liu H., Luo S. (2019). Vitexin induces apoptosis through mitochondrial pathway and PI3K/Akt/mTOR signaling in human non-small cell lung cancer A549 cells. *Biological Research*.

[41] Maneechote C., Palee S., Kerdphoo S., Jaiwongkam T., Chattipakorn S. C., Chattipakorn N. (2019). Balancing mitochondrial dynamics via increasing mitochondrial fusion attenuates infarct size and left ventricular dysfunction in rats with cardiac ischemia/reperfusion injury. *Clinical Science*.

[42] Buja L. M. (2013). The pathobiology of acute coronary syndromes: clinical implications and central role of the mitochondria. *Texas Heart Institute Journal*.

[43] Terui K., Enosawa S., Haga S. (2004). Stat3 confers resistance against hypoxia/reoxygenation-induced oxidative injury in hepatocytes through upregulation of Mn-SOD. *Journal of Hepatology*.

[44] Zhang Y., Wang Y., Xu J. (2019). Melatonin attenuates myocardial ischemia-reperfusion injury via improving mitochondrial fusion/mitophagy and activating the AMPK-OPA1 signaling pathways. *Journal of Pineal Research*.

[45] Zorov D. B., Juhaszova M., Sollott S. J. (2006). Mitochondrial ROS-induced ROS release: an update and review. *Biochimica et Biophysica Acta*.

[46] Chan D. C. (2006). Mitochondria: dynamic organelles in disease, aging, and development. *Cell*.

[47] Muller G., Morawietz H. (2009). Nitric oxide, NAD (P) H oxidase, and atherosclerosis. *Antioxidants & Redox Signaling*.

[48] van der Bliek A. M., Shen Q., Kawajiri S. (2013). Mechanisms of mitochondrial fission and fusion. *Cold Spring Harbor Perspectives in Biology*.

[49] Ong S. B., Hall A. R., Hausenloy D. J. (2013). Mitochondrial dynamics in cardiovascular health and disease. *Antioxidants & Redox Signaling*.

[50] Vásquez-Trincado C., García-Carvajal I., Pennanen C. (2016). Mitochondrial dynamics, mitophagy and cardiovascular disease. *The Journal of Physiology*.

[51] Yang Y., Zhao L., Ma J. (2017). Penehyclidine hydrochloride preconditioning provides cardiac protection in a rat model of myocardial ischemia/reperfusion injury via the mechanism of mitochondrial dynamics mechanism. *European Journal of Pharmacology*.

[52] Yu J., Maimaitili Y., Xie P. (2017). High glucose concentration abrogates sevoflurane post-conditioning cardioprotection by advancing mitochondrial fission but dynamin-related protein 1 inhibitor restores these effects. *Acta Physiologica (Oxford, England)*.

[53] Zhao T., Huang X., Han L. (2012). Central Role of Mitofusin 2 in Autophagosome-Lysosome Fusion in Cardiomyocytes. *Journal of Biological Chemistry*.

[54] Parra V., Eisner V., Chiong M. (2008). Changes in mitochondrial dynamics during ceramide-induced cardiomyocyte early apoptosis. *Cardiovascular Research*.

[55] Hall A. R., Burke N., Dongworth R. K. (2016). Hearts deficient in both Mfn1 and Mfn2 are protected against acute myocardial infarction. *Cell Death & Disease*.

[56] Figueroa-Romero C., Iñiguez-Lluhí J. A., Stadler J. (2009). SUMOylation of the mitochondrial fission protein Drp 1 occurs at multiple nonconsensus sites within the B domain and is linked to its activity cycle. *The FASEB Journal*.

[57] Cellier L., Tamareille S., Kalakech H. (2016). Remote ischemic conditioning influences mitochondrial dynamics. *Shock*.

[58] Disatnik M. H., Ferreira J. C., Campos J. C. (2013). Acute inhibition of excessive mitochondrial fission after myocardial infarction prevents long-term cardiac dysfunction. *Journal of the American Heart Association*.

[59] Veeranki S., Tyagi S. C. (2017). Mdivi-1 induced acute changes in the angiogenic profile after ischemia-reperfusion injury in female mice. *Physiological Reports*.

